# Two new species of the planthopper genus *Tenguna* Matsumura, 1910, with a key to all species (Hemiptera, Fulgoromorpha, Dictyopharidae)

**DOI:** 10.3897/zookeys.669.10105

**Published:** 2017-04-20

**Authors:** Yan-Li Zheng, Lin Yang, Xiang-Sheng Chen, Xu-Qiang Luo

**Affiliations:** 1 Institute of Entomology, Guizhou University; The Provincial Key Laboratory for Agricultural Pest Management of Mountainous Region, Guiyang, Guizhou 550025 P. R. China; 2; 3 School of geography and tourism, Guizhou Education University, Guiyang, Guizhou, China 550018 P. R. China

**Keywords:** Dictyopharidae, distribution, new species, planthopper, taxonomy

## Abstract

Two new species of the genus *Tenguna* Matsumura, 1910, *Tenguna kuankuoshuiensis* **sp. n.**, *Tenguna plurijuga* **sp. n.**, collected from China, are described and illustrated, photographs of the new species are provided together. A key is given to identify all the known species of *Tenguna*.

## Introduction

The planthopper genus *Tenguna* was established by Matsumura in 1910 based on a single species, *Tenguna watanabei* Matsumura, from Taiwan, China. Song and Liang (2007) reviewed this genus and added the second species, *T. medogensis*, from China. In this paper, two new species, *T. kuankuoshuiensis* sp. n., *T. plurijuga* sp. n., are described and illustrated. Photographs of the adults of the new species are presented.

## Materials and methods

The morphological terminology and measurements used in this study follow Yang and Yeh (1994) and Song and Liang (2007). Material examined here is deposited in the Institute of Entomology, Guizhou University, Guiyang, China (**GUGC**). Dry specimens were used for the observations, descriptions, and illustrations. Genital segments of the examined specimens were macerated in boiling solution of 10% NaOH and drawn from preparations in glycerin jelly under a Leica MZ12.5 stereomicroscope. Color pictures for adult habitus were obtained by a KEYENCE VHX-1000 system. Illustrations were scanned with Canon Cano Scan LiDE 200 and imported into Adobe Photoshop CS6 for labeling and plate composition. Terminology of morphology, genital characters, and measurements follow Song and Liang (2013).

The following abbreviations are used in the text:

**BL** body length (from apex of cephalic process to tip of fore wings);

**HL** head length (from apex of cephalic process to base of eyes);

**HW** head width (including eyes);

**FWL** forewing length;

**GUGC** Guizhou University, Guiyang, China.

## Taxonomy

### Tenguna

Taxon classificationAnimaliaHemipteraDictyopharidae

Matsumura, 1910

Figs 1–13 , 14–18 , 19–32 , 33–37

Tenguna Matsumura, 1910: 104; Song and Liang 2007: 59.

#### Type species.

*Tenguna watanabei* Matsumura, 1910 (original designation).

#### Diagnosis.

Genus diagnostic characters: general color green or yellowish green (in death); vertex with median carina distinct and complete, lateral margins sub-parallel at base, slightly sinuate in front of eyes, then gradually narrowing to arrowhead at apex; pronotum with distinct median carina and two obscure lateral discal carinae, elevated only anteriorly; fore femur with one minute, short and blunt spine near apex; aedeagus with a pair of processes apically and phallobase with pairs of membranous lobes apically.

#### Distribution.

Southern China (Guizhou, Hubei, Sichuan, Taiwan, Xizang).

#### Key to the species of the genus *Tenguna* based on males

**Table:** 

1	Vertex narrow and long, ratio of length to width is greater than 2.4	**2**
–	Vertex broad and short, ratio of length to width is not greater than 2.4	**3**
2	Pygofer posterior margin with distinct, posteriorly directed process near apex in lateral view; phallobase with 2 pairs of membranous lobes at apex	***Tenguna medogensis***
–	Pygofer posterior margin with not distinct, posteriorly directed process near apex in lateral view (Fig. 27); phallobase with 3 pairs of membranous lobes at apex (Figs 11–13)	***Tenguna kuankuoshuiensis* sp. n.**
3	Aedeagus with 1 pair of equally long processes apically, processes with apex acute, sclerotized and pigmented. Phallobase sclerotized and pigmented at base, with 3 pairs of membranous lobes at apex	***Tenguna watanabei***
–	Aedeagus (Figs 30–32) with 1 pair of unequally long processes apically, processes with apex acute, sclerotized and pigmented. Phallobase sclerotized and pigmented at base, with numerous membranous lobes at apex	***Tenguna plurijuga* sp. n.**

### Tenguna kuankuoshuiensis sp. n.

Taxon classificationAnimaliaHemipteraDictyopharidae

http://zoobank.org/55EFD65D-ADC5-4DBE-A001-51E98AD62C47

Figs 1–13 , 14–18

#### Measurements.

♂, BL: 13.1–14.1 mm; HL: 1.7–1.8 mm; HW: 1.5–1.6 mm; FWL: 9.9–10.3 mm. ♀, BL: 15.2–16.3 mm; HL: 1.8–1.9 mm; HW: 1.6–1.8 mm; FWL: 12.3–12.9 mm.

#### Diagnosis.

This species is similar to *Tenguna medogensis*, but can be distinguished from phallobase. The former with three pairs of membranous lobes at apex, the latter with two pairs of membranous lobes at apex.

#### Description.

General color green; carinae on cephalic process, frons, pronotum and mesonotum, and parts of veins on forewings, dark green; rostrum with extreme apex blackish; hind tibia with lateral and apex black-tipped spines .

**Figures 1–13.**
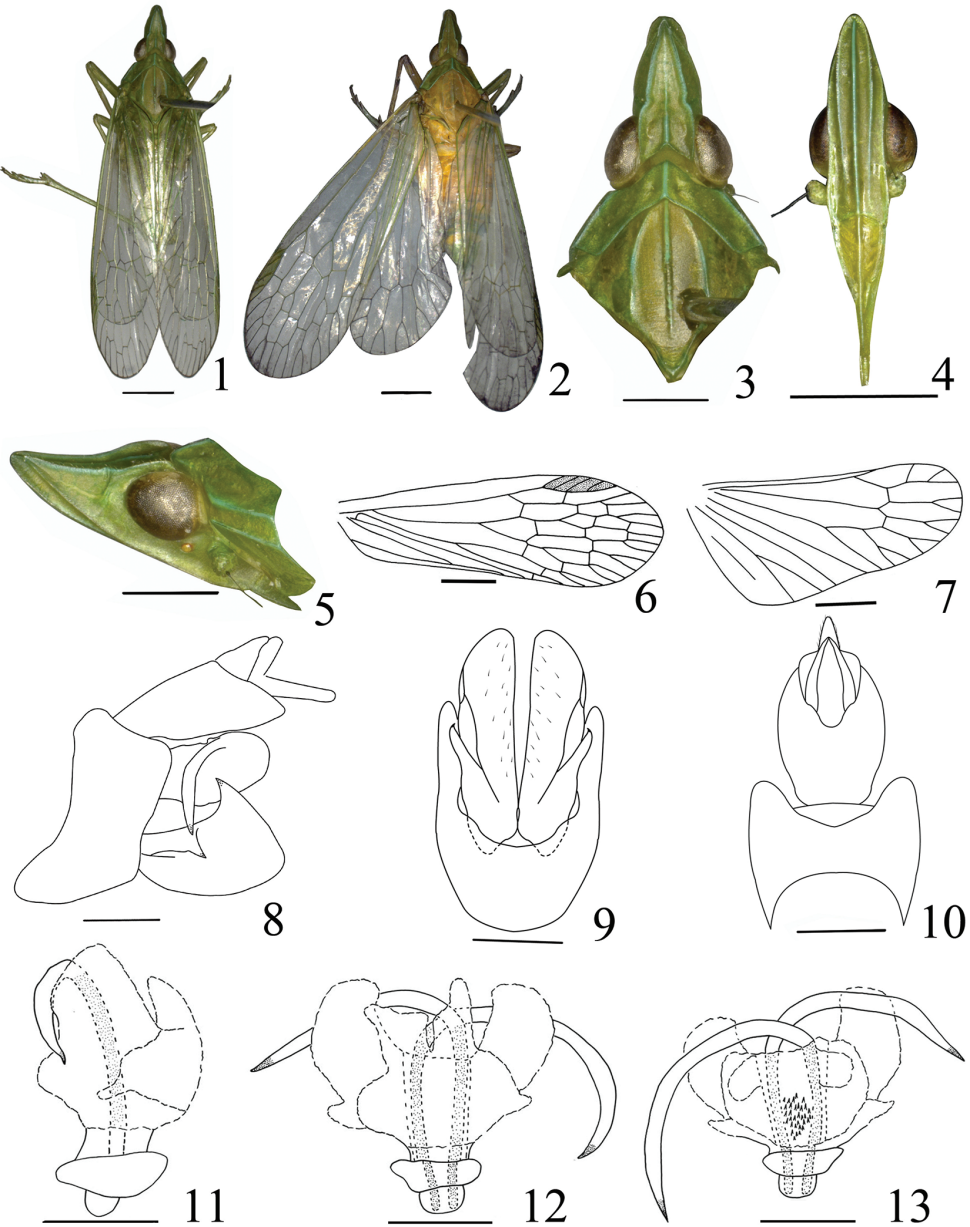
*Tenguna kuankuoshuiensis* sp. n. **1** male, holotype **2** female **3** Head and thorax, dorsal view **4** Frons and clypeus, ventral view **5** Head and pronotum, lateral view **6** Forewing **7** Hind wing **8** Pygofer and anal tube, dorsal view **9** Pygofer and parameres, ventral view **10** Genitalia, lateral view **11** Aedeagus, lateral view **12** Aedeagus, ventral view **13** Aedeagus, dorsal view. Scale bars: **1–7** 2 mm; **8–13** 0.5 mm.

*Cephalic process* (Figs 1–5) relatively short, a little upturned, ratio length to length of pronotum and mesonotum combined 0.6. Vertex (Figs 1–3, 5) with lateral margins carinate, sub-parallel at base, slightly sinuate in front of eyes, then gradually narrowing to arrowhead at apex, ratio of length to width between eyes 2.8. Frons (Fig. 4) elongate, median carina complete and elevated, length 2.6 times long than width. Pronotum (Figs 1–3) distinctly shorter than mesonotum medially in the middle line, median carina distinct, lateral carina obscure, ratio length to length approx. 0.2:1. Forewings (Figs 1, 6) with Sc+R, M and Cu all branched apically; stigma distinct, with 3–5 cells. Legs moderately long; fore femur not flattened and dilated, with one minute, short, blunt spine near apex; hind tibia with 6–7 lateral black-tipped spines and eight apical black-tipped teeth.

*Male genitalia*. Pygofer (Figs 8–10) wider ventrally than dorsally, posterior margin with a blunt process, ventral margin depressed to accommodate anal tube (Fig. 8). Parameres (Figs 8, 9) large, distinctly broadening towards apex in lateral view (Fig. 8), posterior margin straight, upper margin with dorsally directed, black-tipped process near middle, with ventrally directed, hook-like process near sub-middle on outer upper edge. Anal tube (Figs 8, 10) oval in dorsal view, ratio length to width approx. 2.0:1. Aedeagus (Figs 11–13) with one pair of special long endosomal processes, processes with apex acute, sclerotized and pigmented. Phallobase sclerotized and pigmented at base, with three pairs of membranous lobes at apex: the dorsal lobe large and the ventral lobe with small lobe in lateral view (Fig. 11), two pairs of large lobes in dorsal view (Fig. 12), three pairs of lobes and numerous small spines on it in ventral view (Fig. 13).

*Female genitalia*. Anal tube (Fig. 15) round and large in dorsal view, ratio length to width at middle nearly 1.0. First valvula (Fig. 16) sclerotized with six different sized teeth in lateral view. Second valvulae (Fig. 17) triangular, symmetrical in ventral view, connected at base and separated from 1/5 base. Third valvula (Fig. 18) with two sclerotized lobes, lateral lobe with six long spines at apex.

**Figures 14–18.**
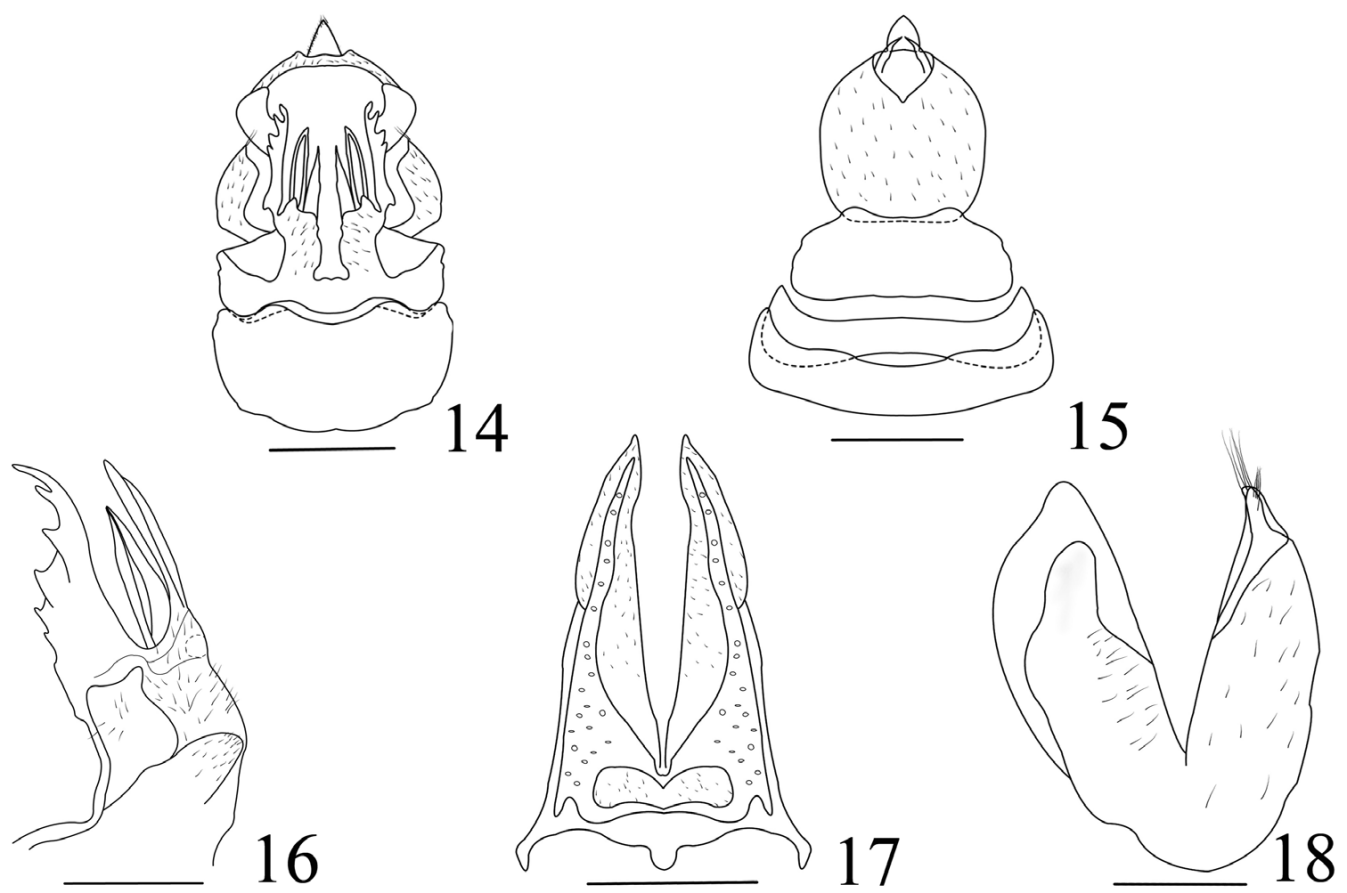
*Tenguna kuankuoshuiensis* sp. n. **14** Genitalia ventral view of female **15** Genitalia dorsal view of female **16** First valvulae (lateral view) **17** Second valvulae (ventral view) **18** Third valvulae (lateral view). Scale bars 0.5 mm.

#### Type material.

Holotype ♂, China, Guizhou, Kuankuoshui, Qing gang tang hui long county. 17.VIII. 2010, Lihu. Paratypes, 1♂2♀♀, same to Holotype; 9♂♂5♀♀, China, Guizhou, Fanjing mountain, 27.VII. 2001, Lizizhong.

#### Etymology.

This new species is named for the holotype occurrence in “Kuankuoshui”, Guizhou province in southwestern China.

### Tenguna plurijuga sp. n.

Taxon classificationAnimaliaHemipteraDictyopharidae

http://zoobank.org/F0E37768-11AC-4887-B16B-ADBED289B40B

Figs 19–32 , 33–37

#### Measurements.

♂, BL: 14.9 mm; HL: 2.1 mm; HW: 1.6 mm; FWL: 11.4 mm. ♀, BL: 15.2–16.7 mm; HL: 2.2–2.5 mm; HW: 1.6–1.8 mm; FWL: 11.6–12.3 mm.

#### Diagnosis.

This species can be distinguished from other species from aedeagus and phallobase. The aedeagus with pair of unequal long processes apically; phallobase with numerous membranous lobes at apex.

#### Description.

General color and external characters as the *Tenguna kuankuoshuiensis* sp. n.

**Figures 19–32.**
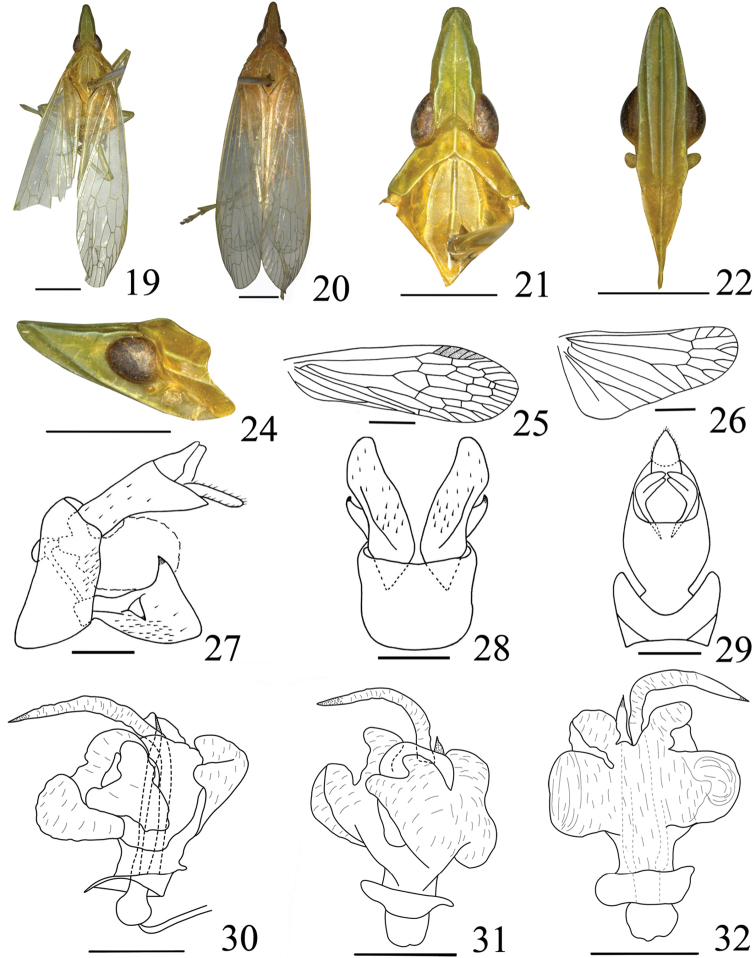
*Tenguna plurijuga* sp. n. **19** male, holotype **20** Same, female **21** Head and pronotum, lateral view **22** Head and thorax, dorsal view **23** Frons and clypeus, ventral view **24** Head and pronotum, lateral view **25** Forewing **26** Hind wing **27** Pygofer and anal tube, dorsal view **28** Pygofer and parameres, ventral view **29** Genitalia, lateral view **30** Aedeagus, lateral view **31** Aedeagus, ventral view **32** Aedeagus, dorsal view. Scale bars **19–26** = 2 mm; **27–32** 0.5mm.

*Cephalic process* (Figs 19–24) relatively short, a little upturned, ratio length to length of pronotum and mesonotum combined 0.7. Vertex (Figs 19–22, 24) with lateral margins carinate, sub-parallel at base, slightly sinuate in front of eyes, then gradually narrowing to arrowhead at apex, ratio of length to width between eyes 2.3. Frons (Fig. 23) elongate, median carina complete and elevated, length 3.0 times long than width. Pronotum (Figs 21, 24) distinctly shorter than mesonotum medially in the middle line, median carina distinct, lateral carina obscure, ratio length to length approx. 0.2:1. Forewings (Figs 19, 20, 25) with Sc+R, M and Cu all branched apically; stigma distinct, with five cells. Legs narrow and moderately long; fore femur with one minute, short, blunt spine near apex; hind tibia with six lateral black-tipped spines and eight apical black-tipped teeth.

**Male genitalia.** Pygofer (Figs 27–29) wider ventrally than dorsally, posterior margin with a blunt process, ventral margin depressed to accommodate anal tube. Parameres (Figs 27, 28) large, distinctly broadening towards apex in lateral view (Fig. 27), posterior margin straight, upper margin with dorsally directed, black-tipped process near middle, with ventrally directed, hook-like process near sub-middle on outer upper edge. Anal tube (Figs 27, 29) oval in dorsal view, ratio length to width 2.0:1. Aedeagus (Figs 30–32) with one pair of unequal long processes apically, processes with apex acute, sclerotized and pigmented. Phallobase sclerotized and pigmented at base, with numerous membranous lobes at apex.

**Female genitalia.** Anal tube (Fig. 34) round and large in dorsal view, ratio of length to width at middle approx. 0.7. First valvula (Fig. 35) sclerotized with seven differently sized teeth in lateral view. Second valvulae (Fig. 36) triangular, symmetrical in ventral view, connected at base and separated from 1/5 base. Third valvula (Fig. 37) with 2 sclerotized lobes, lateral lobe with 4 long spines at apex.

**Figures 33–37.**
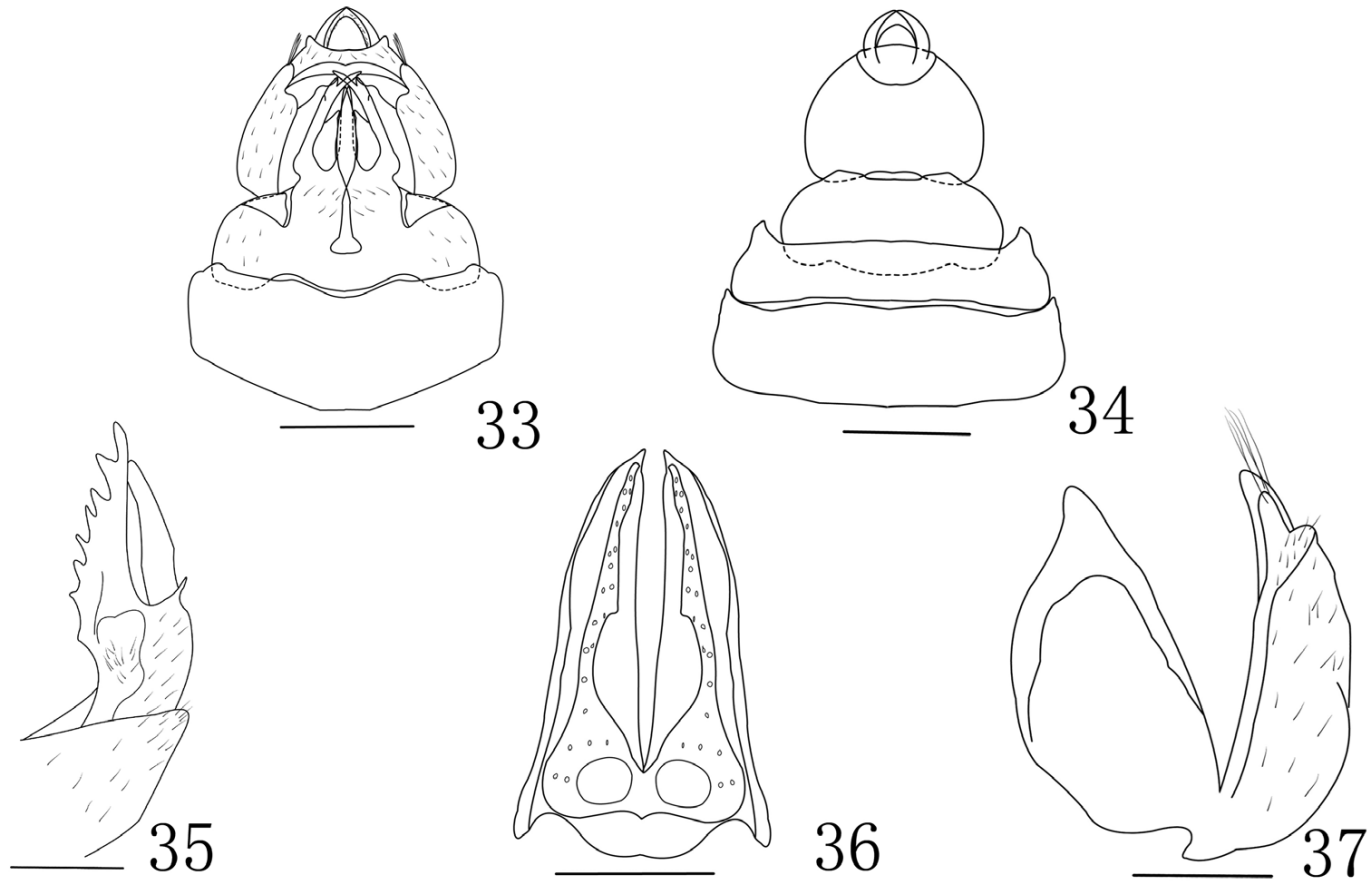
*Tenguna plurijuga* sp. n. **33** Genitalia ventral view of female **34** Genitalia dorsal view of female **35** First valvulae (lateral view) **36** Second valvulae (ventral view) **37** Third valvulae (lateral view). Scale bars 0.5 mm.

#### Type material.

Holotype ♂, China, Guizhou, Institute of Entomology. XII. 2008, Light trap, Zhangyubo. Paratypes, 6♀♀, data same as holotype.

#### Etymology.

This new species is named for the Greek word “*plurijuga*” referring to aedeagus with numerous membranous lobes at apex.

## Discussion

The discovery of two new species broadens our knowledge of the morphology and biogeography of the genus. The two new species both occur in Guizhou, China. This may be due to the climate of Guizhou, warm and humid, subtropical humid monsoon, and minimal temperature changes. All described species are distributed in the Palearctic and Oriental regions.

## Supplementary Material

XML Treatment for Tenguna

XML Treatment for Tenguna kuankuoshuiensis

XML Treatment for Tenguna plurijuga
